# Epigenetic Modifications in Plant Development and Reproduction

**DOI:** 10.3390/epigenomes5040025

**Published:** 2021-11-19

**Authors:** Vladimir Brukhin, Emidio Albertini

**Affiliations:** 1 Plant Genomics Laboratory, ChemBio Cluster, ITMO University, 9 Lomonosova Street, 191002 Saint-Petersburg, Russia; 2 Department of Plant Embryology & Reproductive Biology, Komarov Botanical Institute RAS, 2 Professor Popov Street, 197376 Saint-Petersburg, Russia; 3 Department of Agricultural, Food and Environmental Sciences, University of Perugia, Borgo XX Giugno 74, 06121 Perugia, Italy; emidio.albertini@unipg.it

**Keywords:** epigenetics, plant development, microsporogenesis, megasporogenesis, male and female gametophytes, embryogenesis, endospermogenesis, sexual and asexual reproduction

## Abstract

Plants are exposed to highly fluctuating effects of light, temperature, weather conditions, and many other environmental factors throughout their life. As sessile organisms, unlike animals, they are unable to escape, hide, or even change their position. Therefore, the growth and development of plants are largely determined by interaction with the external environment. The success of this interaction depends on the ability of the phenotype plasticity, which is largely determined by epigenetic regulation. In addition to how environmental factors can change the patterns of genes expression, epigenetic regulation determines how genetic expression changes during the differentiation of one cell type into another and how patterns of gene expression are passed from one cell to its descendants. Thus, one genome can generate many ‘epigenomes’. Epigenetic modifications acquire special significance during the formation of gametes and plant reproduction when epigenetic marks are eliminated during meiosis and early embryogenesis and later reappear. However, during asexual plant reproduction, when meiosis is absent or suspended, epigenetic modifications that have arisen in the parental sporophyte can be transmitted to the next clonal generation practically unchanged. In plants that reproduce sexually and asexually, epigenetic variability has different adaptive significance. In asexuals, epigenetic regulation is of particular importance for imparting plasticity to the phenotype when, apart from mutations, the genotype remains unchanged for many generations of individuals. Of particular interest is the question of the possibility of transferring acquired epigenetic memory to future generations and its potential role for natural selection and evolution. All these issues will be discussed to some extent in this review.

## 1. Epigenetic Systems in Plants

In the last two decades, much data on the epigenetic regulation of plants have appeared, as well as works summarizing the accumulated knowledge [1,2,3,4]; nevertheless, many questions remain unclear, and a number of results are contradictory. New in this area, data are constantly emerging. We tried to take into account and discuss the main findings and conclusions in this field.

Epigenetics, ‘epi’ (upon, above, beyond) and ‘genetic’ (DNA sequence), refers to a layer of information that exists beyond that encoded in the DNA sequence. Therefore, although, in a given organism, the complement of DNA is essentially the same in all somatic cells, patterns of gene expression differ greatly among different cell types, and these patterns can be clonally inherited. Today the term epigenetics also include transient mechanisms separate from the one required to maintain it. Epigenetic regulation of gene expression is understood primarily as DNA methylation, modification of histones by methylation, acetylation, and ubiquitination of histone N-tails, and post-transcriptional silencing through small non-coding RNAs. In the model plant Arabidopsis, more than 130 genes are known that are regulated epigenetically [2].

In plants, 5-cytosine is the main site of DNA methylation. The latter occurs in the three contexts, symmetric CH, CHG, and asymmetric CHH, where H is any nucleotide except G. In the symmetrical context, the most frequently methylated are repetitive motives of CG or CHG, referred to as CG islands. CG methylation during replication cycles is supported by the enzyme methyltransferase 1 (MET1) in a semi-conserved way. Methylation in the context of CHG is supported by chromomethylase 3 (CMT3) methyltransferase. Due to its asymmetric nature, CHH must be methylated de novo after each round of replication. CHH methylation sites are catalyzed by domain-rearranged methyltransferase 2 (DRM2), which is involved in plant-specific RNA-directed DNA methylation (RdDM). CHH methylation can also be performed by chromomethylase 2 (CMT2) homologous to CMT3 independently of RdDM. The enzyme decrease in DNA methylation 1 (DDM1) remodels chromatin by removing the histone H1 linker in the compact heterochromatic regions, providing access to methyltransferases to DNA. DRM2-mediated methylation is mainly involved in the methylation of euchromatic regions, including short transposable elements (TE) and edge fragments of long TE, as well as pericentromeric sites [5].

An important role in the biogenesis of small interfering RNAs is played by polymerase IV and polymerase V, which are plant homologs of polymerase II, and that specialize in the production of small RNAs required for RdDM. Polymerase IV synthesizes single-stranded RNA (ssRNA) on silencing targets, retrotransposons, viruses, transgenes, or repetitive genes. RNA-dependent RNA polymerase 2 (RDR2) promotes the formation of double-stranded RNA (dsRNA) from ssRNA. Next, diser-like 3 (DCL3) cuts dsRNA into 24- and 23-nt small interfering RNAs (siRNAs), one strand of the duplex are loaded to Argonaute (AGO4). AGO4-bound siRNAs complement with polymerase V transcripts and recruit DRM2, which catalyzes de novo methylation of the genome homologous sites in all contexts [6,7].

Active DNA demethylation occurs via DNA glycosylase repressor of silencing 1 (ROS1), demeter (DME), demeter-like 2 (DML2), and DML3. The mode of action of glycosylases is through the elimination of methylated cytosine, replacing it with non-methylated cytosine. During plant reproduction and further ontogenesis, various modes of dynamic DNA methylation and demethylation in all contexts provide the necessary genetic regulation of development [8].

In addition to DNA methylation, the modification of histones, nuclear proteins involved in the packaging of DNA strands in the nucleus and in the epigenetic regulation of transcription and replication is of great importance for genetic regulation. Chromatin remodeling (a change in its structure) occurs, among other things, due to histone modifications. It alters the availability of DNA for transcription factors and polymerases, thereby regulating gene expression and contributing to phenotype variability. Like DNA methylation, chromatin remodeling plays an important role in plant reproduction and ontogenesis. Basically, the epigenetic marks are the methylation of lysines in histone H3. Thus, the trimethylation of lysine 3 and lysine 4 in histone H3 (H3K4me3 and H3K3me3, respectively) leads to the formation of “active” chromatin, which allows genes to be expressed, while dimethylation of lysine 9 and trimethylation of lysine 27 (H3K9me2 and H3K27me3, respectively) produces repressive chromatin, which suppresses the transcriptional activity of genes. The formation of repressive chromatin containing H3K27me3 regulates the evolutionarily conserved complex of proteins polycomb repressive complex 2 (PRC2), which regulates many developmental processes of reproduction and the initial stages of seed formation in plants.

Histone acetylation is usually an epigenetic mark associated with active chromatin and transcriptional activity [9].

## 2. Methylation in the Meristem Development

A distinctive feature of plant morphogenesis is the totipotency of some cells of plant meristems, which perform the function of stem cells. These cells can transform into any cell of plant tissue or organ. Since meristems persist throughout the whole life of a plant, new organs and tissues can form indefinitely, and in this sense, plants are immortal. In other words, clones of plant genotypes can exist indefinitely through vegetative propagation, as well as by in vitro propagation in tissue culture. In plants, in addition to the shoot apical meristem, which gives rise to leaves, stem, and flower buds, there are also lateral, intercalary, and marginal meristems, whose cells, under certain conditions, are capable of organogenesis and somatic embryogenesis. In Waddington’s terms, meristematic totipotent plant cells are polyvalent and can follow many trajectories of the epigenetic landscape [10,11].

**Figure 1:**
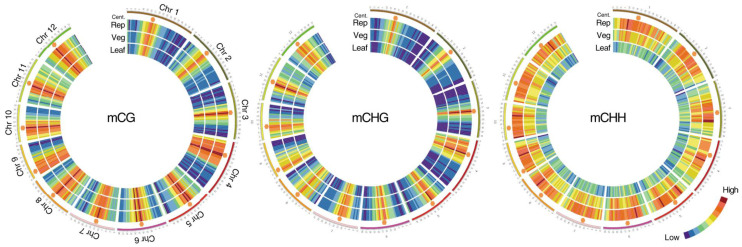
Methylation in the vegetative and reproductive SAMs. Heat maps showing cytosine methylation levels in the 12 rice for the CG (**left**), CHG (**middle**), and CHH (**right**) contexts. SAM (Veg)—vegetative meristem, SAM (Rep)—reproductive meristem, (Leaf)—leaf. Orange hexagons mark pericentromeric regions. Modified from the work of [12].

During the transition from the vegetative to the generative phase of development in the shoot apical meristem (SAM), the patterns of DNA and histone methylation change significantly (Figure 1) [12]. After epigenetic modifications occur in the stem cells of the meristems due to gradients of phytohormones and interaction with the environment, the shoot apical meristem turns into a flower meristem, in which ovary formation and meiosis take place. Unlike animals, plants do not form germ lines directly; instead, they form haploid male and female gametophytes in which gametogenesis occurs. Since male and female gametophytes are haploid, possible harmful mutations that have arisen are not compensated by the homologous allele, and gametophytes carrying such mutations are aborted [13]. However, zygotic embryoletal and sporophytic mutations can pass through the gametophyte stage since their function becomes noticeable only at the zygote/embryo and sporophyte (diploid plant) stages, respectively. These mutations can be transmitted heterozygous in segregated populations [13]. Due to the peculiar gametogenesis and embryogenesis in plants, many epigenetic marks associated with the suppression of transposons are temporarily suppressed by small non-coding RNAs of neighboring tissues [14,15].

**Figure 2:**
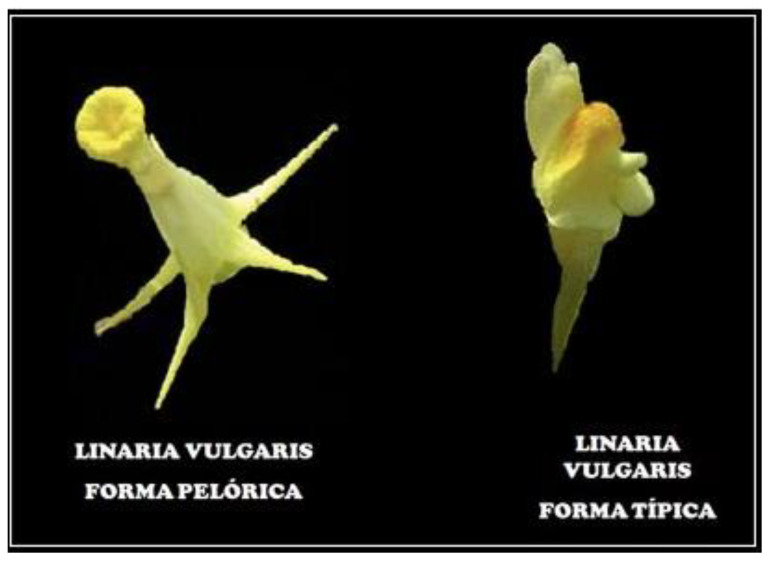
Actinomorphic (peloric) and zygomorphic (most typical) flowers in toadflax (*Linaria vulgaris*). The symmetry and shape of the flower are determined by the methylation of the *Lcyc* gene.

Perhaps the first epiallele in nature was found in toadflax (*Linaria vulgaris*) when Karl Linnaeus studied the shape of the flower. In wild-type plants, the flower shape is zygomorphic, that is, bilaterally symmetric, while several peloric flowers develop radial symmetry, i.e., become actinomorphic (Figure 2). Genetic analysis showed that radial symmetry arises because of methylation of the *Lcyc* locus, while transcripts of this gene are not found in actinomorphic flowers [16]. *Lcyc* is homologous to the *Cycloidea* gene, which controls the symmetry in the snapdragon flower [17].

Another well-known example of epiallele is the *CNR* (Colorless non-ripening) locus in tomatoes [18]. In the mutants with this epiallele, seed maturation is suppressed, and colorless mealy fruits are formed. Genetic analysis showed that in individuals containing the mutant allele, the SBP transcription factor is poorly expressed due to the high methylation of the upstream region of the *Le SPL-CNR* gene.

Epimutations occur much more often and faster than proper mutations. However, unlike the latter, epimutations are reversible and thus are of great importance in the quick tuning and plasticity of the phenotype. Epigenetic diversity can provide phenotypic variability, which is valuable both for the fitness of the plant populations to changing environments and could be used for breeding agricultural plants.

## 3. Epigenetic Regulation of Microsporogenesis and Male Gametophyte Development

As already mentioned, in plants, primary germ cells do not directly enter spermatogenesis and oogenesis, in contrast to animals. Instead, in the flower meristem, pollen mother cells (PMCs) in anthers and megaspore mother cells (MMCs) in ovaries are formed as a result of two meiotic divisions followed by the series of mitotic divisions producing haploid male and female gametophytes, where male and female gametes origin, respectively [17,18]. In angiosperms, male and female gametophytes, despite their small size and small number of cells, are excellent models for studying morphogenesis and epigenetic control of cell growth and specialization, cell polarity, and signaling processes.

In the case of pollen development, which occurs in the anthers, two stages can be distinguished, microsporogenesis and microgametogenesis. Diploid microsporocytes, or PMCs, are formed in the sporogenous layer of the anther. Two meiotic divisions of PMC produce tetrad of four haploid cells. Then tetrads separate into individual microspores. Afterward, two mitotic divisions take place: in the first asymmetric cytokinesis forms a large vegetative and a smaller generative cell, followed by the generative cell division producing two sperm cells, while the vegetative cell does not divide anymore. So, mature pollen grain consists of three cells, a larger vegetative cell, which controls and implements the growth of the pollen tube during fertilization, and two smaller sperm cells, which take part in double fertilization [19].

Epigenetic rearrangements play an important role in the regulation of both male and female gametophytes development, as well as in fertilization [14,20,21,22,23].

In Arabidopsis, it has been shown that in the PMC methylation level in a symmetric context, CG and CHG, was higher compared to asymmetric context, CHH [20]. It is known that symmetric methylation occurs mainly in transposable elements, while asymmetric hypermethylation usually takes place in protein-coding genes. It is likely that increased methylation in a symmetric context promotes suppression of TE activity, which ensures genome stability before and during meiosis. At the same time, inactivation of methylation in an asymmetric context promotes the activation of genes necessary for launching sperm cell formation programs and further fertilization. In addition to DNA methylation, during the maturation of PMCs, a dramatic reorganization of chromatin occurs, which promotes the commence of meiosis. The change from the mitotic to the meiotic phase is accompanied by an increase in permissive chromatin (H3K4me3) and a reduction in repressive chromatin (H3K27me1 and H3K27me3) [24]. After meiosis and asymmetric mitotic division of the haploid microspore, the vegetative cell becomes roundish. It has an increased level of methylation in the CHH regions, while it significantly loses the centromere-specific histone H3 (CENH3) because of the decondensation of pericentromeric heterochromatin, local hypomethylation due to the activity of DME/ROS1 demethylases, and activation of transposable elements is also observed [14,15,25]. The centromeric histone H3 (CENH3) variant plays an important role in the assembly and function of kinetochores during mitotic and meiotic cell division. Kinetochore assembly begins with the incorporation of CENH3 into centromeric nucleosomes. The deposition of CENH3 on centromeres varies with the stage of the cell cycle. CENH3 is also essential for vegetative cell division and further DNA elimination [26]. Hypomethylation of TEs results in the generation of 21–22 nt siRNAs, which are transported to the sperm cells to suppress their TEs by RdDM methylation [14]. In general, whole-genome cell-specific methylation profiling revealed a high level of CG and CHG methylation in the DNA of microspores, sperm, and vegetative cells during the entire period of pollen formation and development. While most of the CHH methylation is lost in the pericentromeric region of microspores and sperm cells, it is restored in the vegetative cells.

## 4. Epigenetic Regulation of Megasporogenesis and Female Gametophyte Development

**Figure 3:**
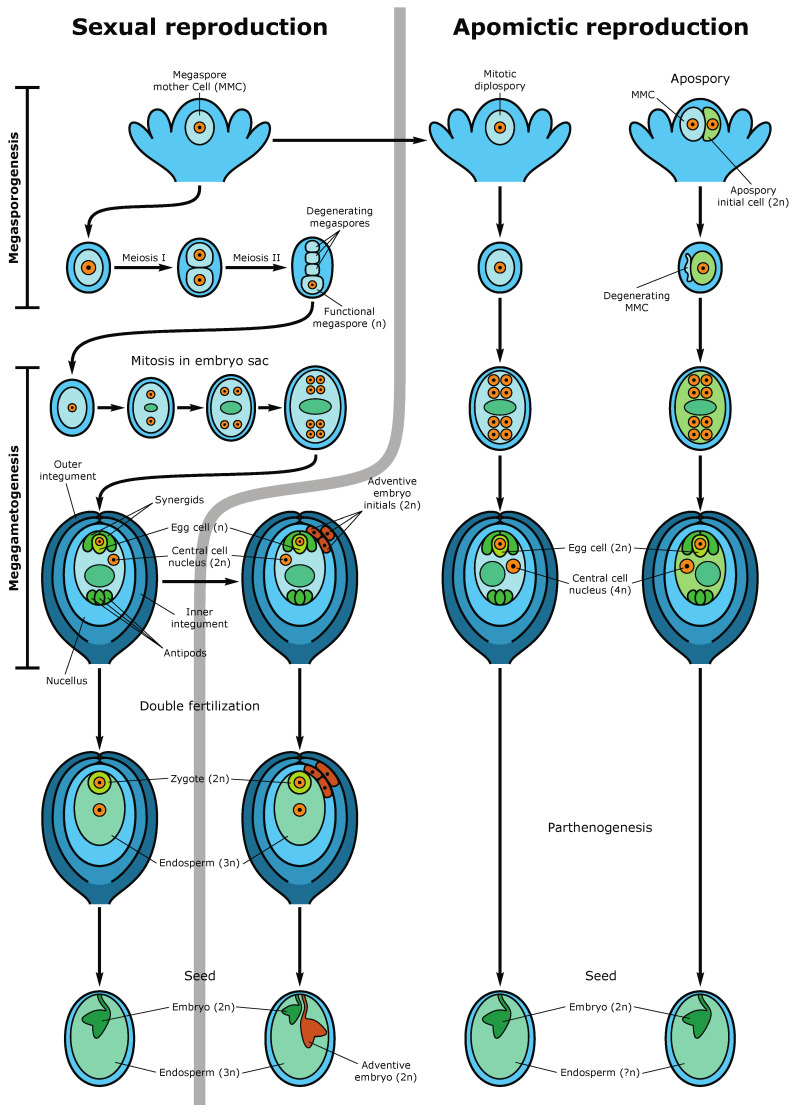
Sexual and apomictic seed development. The left side demonstrates the sexual pathway and sporophytic apomixis (adventive embryony). In the sexual pathway, the megaspore mother cell (MMC) undergoes two meiotic divisions producing a tetrad of haploid megaspores. Three megaspores degenerate while the functional megaspore gives rise to the embryo sac (female gametophyte) following three rounds of mitotic divisions. The mature embryo sac consists of 7-cells (8 haploid nuclei) in which the egg cell and two synergid cells are located at one pole of the embryo sac and three antipodal cells at the opposite pole. Two polar nuclei fuse to form the diploid nucleus of the central cell. During double fertilization, one sperm fertilizes the egg cell, forming the diploid zygote and the second sperm fertilizes the central cell producing the first nuclei of the triploid endosperm. Synergids participate in the perception of the sperm cells and burst after fertilization. The mature seed consists of the diploid embryo, the triploid endosperm (nourishing tissue), and the seed coat. An adventive embryo may develop from the nucellar or inner integumental sporophytic tissues and develops alongside the sexual embryo. The right part of the figure shows gametophytic apomixis. Meiosis and chromosome reduction are missing. Embryo sac development is initiated from an unreduced MMC, which gives rise to not haploid tetrads but diploid cells of the dyad (diplospory), or from an apospory initial cell (apospory). The embryo develops parthenogenetically from the unreduced egg cell while the endosperm is formed either autonomously or through fertilization of the central cell (in the case of pseudogamy). The mature apomictic seed contains the diploid embryo, and the endosperm ploidy may vary but usually not less than 4n [27].

The formation of an angiosperm egg cell begins with the development of archesporial cells from the subepidermal layer of the flower bud’s nucellus. Archesporial cells produce a diploid dense cytoplasmic, large nuclear megaspore mother cell (MMC), or megasporocytes. MMC undergoes two meiotic divisions, forming a tetrad of megaspores, one of which is referred to as functional megaspores, passing through three rounds of mitosis, produces an eight-nuclear megagametophyte, also called an embryo sac. After cellularization, the embryo sac becomes seven cellular: at one pole, there is an egg apparatus, consisting of an egg cell and two synergids, the latter contribute to the attraction of the sperm cell and fertilization, and at the other pole, there are three antipodal cells that take part in the nutrition of the embryo sac [13,27]. In the center of the embryo sac, there are two polar nuclei, which as a result of karyogamy, fuse to form the homodiploid nucleus of the central cell. During fertilization, the pollen tube enters the embryo sac in the egg apparatus area, and one sperm fertilizes the egg cell, forming a diploid zygote, from which the embryo (new sporophyte) then develops, and the second sperm fuses with the central cell nucleus, producing a triploid nucleus of the first endosperm cell (nourishing tissue for the embryo) (Figure 3). This process is called double fertilization.

During megasporocyte formation (MMC), the level of DNA methylation temporarily decreases in the context of CHH, but methylation in the CG context remains basically unchanged [21]. The specification and differentiation of the MMC, as well as the functional megaspore, is carried out, inter alia, through intercellular interactions by the mobile trans activating siRNAs (tasiRNAs) produced in the surrounding cells of nucellus and transported to the MMC, where they implement silencing at the transcriptional and translational level [28]. It was shown in Arabidopsis that the formation of such siRNAs is regulated by AGO9, RDR6, and SDS3 (a suppressor of genetic silencing 3) enzymes [29]. Disruption of AGO5 expression in Arabidopsis nucellus impairs the initiation of megagametogenesis.

Methylation in the CG and CHH contexts remains stable throughout megagametogenesis. At the same time, as was found in Arabidopsis, CG methylation within genes and transposons of the central cell of the embryo sac was lower than that in sperm cells [30]. Demethylation of DNA in the central cell was performed by the demeter activity [31]. This may indicate that the potential transcription of male genes is suppressed even before fertilization. During gametogenesis in the embryo sac, epigenetic regulation takes place by mobile non-coding tasiRNAs, so siRNAs from the central cell enter the egg cell and suppress the activity of transposable elements. The foregoing confirms the importance of epigenetic control in megasporgenesis and megagametogenesis.

## 5. Role of Epigenetics in Fertilization, Embryogenesis, and Endospermogenesis. Imprinted Genes

Fertilization abolishes CHH hypomethylation of the paternal genome both in the embryo and in the endosperm [32]. It is likely that remethylation of the paternal genome occurs via maternal siRNAs [33]. One of the reasons for the epigenetic suppression of the male genome at the early stages of embryogenesis might be maternal control of the size of the embryo and endosperm [22], as well as the recognition of self-pollen, which can be critical in interspecific crosses [34].

Proper and consistent methylation of the dividing egg cell genome is highly essential for normal embryo development. Young embryos and endosperm tissues are hypomethylated in comparison with mature embryos, which reflects the high transcriptional activity of genes in the developing embryo and preparation for the dormancy of the mature embryo [35,36]. However, during seed germination, the metabolic and, accordingly, transcriptional-genetic activity of the embryonic tissues increases again, which is accompanied by a decrease in the methylation level in the CHH context, which is associated with the activation of protein-coding genes expression [37].

Since the histones inherited from the egg and sperm are not reproduced in the cells of the embryo but are synthesized anew, the epigenetic “memory” associated with the histone marks is eliminated and is not passed to the next generations of cells [37]. Thus, embryogenesis is the second after meiosis checkpoint or clearing box, which removes genome epigenetic marks acquired by the maternal sporophyte. Compared to the embryo, the level of endosperm methylation is significantly lower, which reflects its high transcriptional and metabolic activity. At the same time, the paternal genomes (i.e., genomes introduced by sperm cells into the egg cell and the central cell of the embryo sac) of the embryo and endosperm are more methylated than maternal ones [31]. Apparently, endosperm demethylation is also necessary to suppress the activity of transposable elements through the formation of siRNAs, which are transported into the cells of the adjacent embryo and, through RNA-directed DNA methylation, methylate the terminal regions of the transposons, thereby inactivating them [23].

An important role in endosperm formation plays the histone lysine transferase proteins, which are members of the two groups polycomb (PcG) and trithorax (TrxG), which are involved in the modification of histones due to methylation of lysines in histone tails. They maintain a transcriptionally suppressed and transcriptionally active state, respectively [38]. Proteins containing polycomb repressive complex 2 (PRC2) are required at all stages of plant development and are under strict genetic control, mainly through DNA demethylation. The main function of PRC2 in the central cell is to suppress its proliferation prior to fertilization. In Arabidopsis, PRC2 consists of the four main components (subunits): MEA, FIS2, FIE, and MSI1. The genes encoding these subunits express in the central cell of the embryo sac, and mutations in any of these genes cause the development of endosperm independent of fertilization, i.e., autonomous development [33,39,40,41]. In addition, mutations in the genes encoding the PRC2 subunits are imprinted. That is, they demonstrate a parental of origin effect; therefore, only alleles from one parent are expressed. For example, mutants *mea* and *fis2* demonstrate a maternal effect, accompanied by abnormal seed development and excessive size of the embryo and endosperm, and embryo lethality is independent of the paternal contribution and gene dosage [42,43]. During normal development, only the maternal alleles of *MEA* and *FIS2* are expressed, and their activation is mediated by DME demethylation [43,44].

Thus, in the course of sexual embryogenesis, there is a secondary, after meiotic events, erasure of epigenetic marks occurs, so that methylation of DNA and histones is almost completely de novo. The level of methylation in the embryo is higher than in the endosperm, and it continues to increase in both symmetric and asymmetric contexts as the embryo matures, which is necessary for stabilization of the genome by neutralizing mobile genetic elements, as well as for a gradual decrease in the activity of protein-coding genes to prepare the embryo for seed dormancy. Since the endosperm is metabolically active during the development of the seed, it partially or completely disappears in the mature seed, being absorbed by the tissues of the embryo, there is no need for increased methylation of its genome. An important role in the development of the embryo and endosperm is played by the proteins of the PRC2 complex, which methylates lysine K27 in histone H3 tail and thereby maintains the suppression of gene transcription. In addition, the PRC2 complex promotes the asymmetric expression of some genes from the parental alleles (imprinting), which is necessary for the normal development of the seed. Epigenetic regulation occurs somewhat differently during the asexual reproduction of plants.

## 6. Epigenetic Inheritance by the Next Generations. Apomictic and Asexual Reproduction

Sustainable inheritance of epigenetic marks and their preservation in future generations is an important condition for the acquisition of stable, selective advantages by a plant population. As we discussed earlier, DNA demethylation occurs during meiosis and early embryogenesis, while de novo methylation takes place at the late stages of embryogenesis and continues throughout the entire subsequent ontogenesis of a new sporophyte. As for chromatin, the inheritance of its modifications is a rather complicated process. As one knows, during meiosis and after fertilization, histones are formed anew and, thus, epigenetic marks of histone tails are not transmitted from the previous generations. Nevertheless, there is putative crosstalk between DNA methylation and chromatin remodeling. In this regard, given that the cytoplasm of the egg cell is inherited from MMC, some transmission of epigenetic information about the modification of histones by the RdDM mechanism is possible, bearing in mind that small non-coding RNAs are transferred from the MMC cytoplasm.

Despite the demethylation that occurs during meiosis and embryogenesis, some methylation patterns in the CG context are quite stable and can pass through meiosis and be inherited by the next generations [45,46]. However, methylation in the CHH and CHG contexts is more sensitive to meiotic resetting of methylation [47].

As shown in Arabidopsis, spontaneous nucleotide mutations are extremely rare, numbering 20 SNPs per genome after 30 plant generations originated from one plant, while new methylation was found in thousands of cytosines [45,46]. However, regular changes in methylation in large regions of the genome in the same series of generations were also rarely found. That is, the homeostasis of methylation maintenance was quite stable due to the coordinated functioning of methylases, methyltransferases, and demethylases. There is an assumption that the emergence of new alleles is associated with differential methylation of transposons, which play a major role in the origin of new epialleles and their inheritance [47].

One of the striking examples of transgenerational inheritance of epigenetic modifications is the study of the acquisition of inherited methylation of some Arabidopsis genes caused by water stress [48]. In this study, low relative humidity caused DNA methylation at two loci responsible for stomatal formation, accompanied by the synthesis of large amounts of small RNAs associated with this methylation. Acquired methylation appeared in several generations of offspring but was reversible if the stress leading to the methylation was not repeated.

In the case of asexual reproduction, i.e., via vegetative parts of plants such as shoots, stolon, tubers, runners, rhizomes, etc., and via somatic embryogenesis in tissue culture development of gametophytes is bypassed; therefore, complete demethylation and deletion of histone marks do not occur. Thus, epigenetic patterns can be partially or completely transferred to the next generations of sporophytes. Accordingly, the epigenetic variability in reproductive and asexual plants is different. However, there is no suppression of transposon activity due to methylation by means of tasiRNA expression in the tissues surrounding the somatic embryo or the initial vegetative bud. This can lead to the mobilization of transposable elements and the emergence of new genotypes called somaclonal variants.

Apomixis is the reproduction via asexual seeds (Figure 3). It appears due to the alternative expression of the genes responsible for sexual reproduction. Most of the apomictic plants are polyploids and highly heterozygous because they came to existence as a result of sophisticated hybridization. So, the epigenetic events, which affect polyploidization facilitate the transition from sexual to apomictic reproduction [27]. Apomixis promotes a wider geographical distribution of the species within agamic complexes and isolates polyploids, helping the latter to preserve and reproduce in these complexes on a par with diploid sexuals. Apomictic reproduction is always facultative, which is due, among other things, to the reversibility of many epigenetic processes. The ability to return to sexual reproduction allows apomicts to participate in speciation and evolutionary processes. In apomicts, meiosis in nucells is either blocked during the first meiotic division, in the case of diplospory, or bypassed and replaced by apomeiosis resulting in apospory [49]. In any case, in diploid apomicts, a diploid embryo sac is formed, which morphologically is like the sexual embryo sac. In an apomictic embryo sac, the diploid egg cell is genetically identical to the maternal sporophyte. It gives rise to a parthenogenetic embryo, which develops without fertilization [27]. Endosperm, on the other hand, develops from a tetraploid central cell either autonomously, i.e., without fertilization, or pseudogamously, that is, after fertilization with a haploid or diploid sperm cell, depending on whether meiosis occurs in the anthers or not. As a rule, meiosis in anthers takes place, and pollen is usually haploid, the same as in sexual plants. Since during apomixis, as well as during vegetative reproduction, meiosis, which removes epigenetic modifications of the genome, is absent, the epigenetic mechanisms in apomicts act differently, and epigenetic marks can be transmitted from generation to generation. Despite the lack of genetic variability, apomicts perfectly adapt to changing environments and can occupy huge habitats. In addition to clonal fixation of the genome, which includes combinations of advantageous traits, success in the spread of apomicts is also ensured by epigenetic regulation, which largely determines the plasticity of their phenotypes and their high fitness to various environmental conditions. Since in the formation of clonal embryos, there is no meiosis, which in the reproductive ovules performs the function of a clearing box, epigenetically formed apomictic genotypes can have transgenerational stability. Thus, epigenetic variants of apomicts perform the function of genetic variants of sexuals, providing fitness and the evolutionary potential of asexual offspring [1]. Epigenetic pathways appear to be involved in restricting sexual female gametophyte formation to a single cell in both *Arabidopsis* and maize [28,50,51,52,53,54,55,56]. In particular, nucellar cells in *ago9* mutants failed to undergo programmed cell death but instead initiated developmental processes reminiscent of apospory. While the apospory-like structures failed to develop further, this study provided evidence that epigenome-level regulations are important in directing female germ cell development. In maize, the loss-of-function *ago104* (a homolog of *AGO9*) mutant produced apomixis-like phenotypes that gave rise to 70% functional unreduced female gametes [54,57].

Another important mechanism involved in the regulation of apomixis and epigenetic rearrangements is oxidative stress. The latter has been proposed as central to sex/apomixis switching in facultatively and cyclically apomictic eukaryotes [58,59]. Several lines of evidence suggest that transitions during reproduction and early seed development are epigenetically regulated by dynamic changes in chromatin state [28,49,53,54,55,56]. Moreover, several pieces of evidence indicate that the DNA methylation pathway acts upon germline or germline-associated cells. In fact, apomeiosis-like phenotypes were induced in reproductive cells by deregulation of DNA methylation [50], and treatments of *Boechera* pistils resulted in a high frequency of conversions from the *Taraxacum*-type diplospory to normal meiotic tetrad formation [60].

Random (stochastic, non-directed) genetic modifications occur, such as genetic drift and modifications to adapt to the changing environment. Random epigenetic mutations can also participate in the formation of adaptive phenotypes through natural selection, provided they are transgenerational. Environment-directed epigenetic modifications arise in a targeted manner due to the interaction of the genotype with the environment; they provide the plasticity of the phenotype [61]. Since the genotype in an asexual population is unchanged over a long series of generations, in the case of some ancient apomicts, sometimes thousands of years, their epigenetic regulation can turn on and off the expression of individual genes and gene families in response to environmental signals. This ensures the plasticity of phenotypes in asexual plants. Because in apomicts, the same genotype is transmitted to many generations, it must be resistant to a wide range of environmental changes; therefore, clonal genotypes have high mean fitness [62]. Accidental methylation can occur either due to errors of the methylation enzymes or unusual environmental conditions. In some cases, stochastic methylation can cause persistent epimutation, which can be fixed by natural selection and passed on to subsequent generations, resembling a normal mutation in DNA sequences. However, as a rule, epimutations provide short-term adaptations to fluctuating environmental conditions. As shown above, the adaptive value of epigenetic variability in reproductive and apomictic plants is different.

During the last decade, several studies have been carried out on apomictic species with the aim of identifying epigenetic changes related to the fate of the reproductive program [49]. The apomixis locus resides in heterochromatic regions rich in transposons both in *Paspalum simplex* and in *Pennisetum squamulatum* [63,64]. In *Eragrostis curvula*, sexual and apomictic genotypes produced differentially expressed TE-related sequences [65], and similar differences were noted for apomictic plants obtained by chromosome doubling [65]. Podio et al., (2014) [66] reported a decrease in parthenogenesis in a natural apomictic *P. simplex* genotype after general artificial genome demethylation.

In *Hieracium pilosella*, ref. [51] hypothesized that sexual cues enabling meiotic tetrad formation in ovules needed for aposporous initial cell formation are hormonal or epigenetic. Recently, in *Paspalum rufum*, ref. [67] showed that several genes, previously associated with reproductive development, reproduction, and apomixis, are affected by developmental stage-specific epigenetics marks.

**Figure 4:**
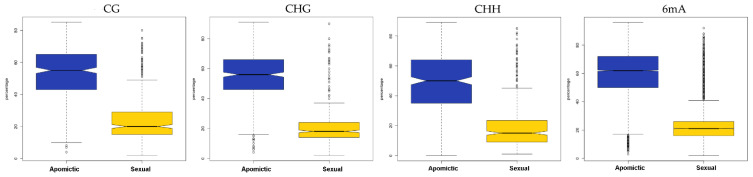
In *E. curvula,* the relative methylation frequencies of the loci contained in each differentially methylated region (DMR) identified from the comparison between the apomictic and sexual plants were hierarchically clustered. The boxplots with 3 samples collapsed for each genotype show that the total number of DMRs for all the four contexts (CG, CHG, CHH, and 6 mA) was higher in the apomictic (blue) than in the sexual (yellow) genotypes (modified from the work of [68]).

In *E. curvula,* Carballo et al., 2021 [68] showed that apomictic genotypes had higher DNA methylation levels than the sexual ones reinforcing the hypothesis that the genes controlling the sexual pathways are present but repressed in apomictic plants (Figure 4).

Owing to advances in modern molecular biology, such as various types of sequencing, including DNA sequencing in a single cell, detection of methylation, protein-protein interaction techniques, cell sorting, and other methods, many aspects of DNA methylation, histone modification in chromatin alteration, and the role of small RNAs in the regulation of plant development and interaction with the environment were studied. Of particular interest is the study of these processes in PMC and MMC during meiosis, gamete maturation, as well as during fertilization, embryogenesis, and further plant development. However, scientists have yet to solve many problems of epigenetics and address important questions: what is the mechanism of “decision making” for DNA methylation and modification of histones in a particular cell of a multicellular organism, how epigenetic homeostasis is maintained, and why is it changed during the certain developmental processes, what is the impetus for changes? An important issue is the transgenerational transmission of DNA and histone methylation and their populational role. The mechanism and the original cause of the asymmetry of epigenetic changes in the parent of origin modifications, that is, imprinting, also remains largely a mystery. Finally, the study of the adaptive value of epigenetic variability in reproductive and apomictic plants requires further research. Of course, intriguing discoveries and answers to many of the questions posed are waiting for us in the near future.
